# Clinic—Traumatic Tetanus

**Published:** 1892-01

**Authors:** J. O’Leary


### CLINIC—TRAUMATIC TETANUS.

A mule was brought to the clinic some time ago affected with lameness of the left foreleg. On examination a foreign body was found imbedded in the frog. This was extracted, and an abundant discharge of ash-colored fœtid pus took place. In a few days the lameness had entirely disappeared, but within a fortnight the animal was brought back presenting the following symptoms : Loss of appetite, ears erect, and immobile eyes fixed and prominent, nostrils dilated, anterior lip contracted, neck stiff and curved, and testicles retracted. Tetanus was diagnosed, the cause being the wound in the hoof. This was examined and the wound found to entirely cicatrised.

The animal was placed in a well ventilated stable and covered with blankets. Aromatic vapor baths were ordered and such food as could be easily masticated. The tetanic symptoms soon became general, with pronounced trismus. No drugs were prescribed, and before the end of the month the animal had entirely recovered under this expectant treatment.

Pathologically, the case is of little importance, but practically, it teaches that diseases often disappear, driven out by organic forces—without the aid of drugs—and it proves that no case is hopeless, even after having been given up by the best veterinarians. —*J. O'Leary, from Gaceta Medico-Verterinaria, Madrid.*

